# The Use of Social Media and mEMA Technology in Comparing Compliance Rate Among Users

**DOI:** 10.31372/20180304.1019

**Published:** 2018

**Authors:** Reimund Serafica, Nada Lukkahatai, Brendan Tran Morris, Kelly Webber

**Affiliations:** a University of Nevada, Las Vegas, School of Nursing, Las Vegas, NV, USA; b Johns Hopkins University, School of Nursing, Baltimore, MD, USA; c Department of Electrical and Computer Engineering, University of Nevada, Las Vegas, Las Vegas, NV, USA; d University of Nevada Cooperative Extension, Reno, NV, USA

**Keywords:** social media, mEMA, dietary self-management, compliance rate

## Abstract

Compliance can be defined as the extent to which a person’s behavior corresponds with agreed recommendations. Within the context of lifestyle intervention, this may refer to physical activity, diet modifications, or lifestyle intervention program attendance or attrition. For lifestyle intervention programs to be successful (as measured against a variety of health and lifestyle markers), it is crucial for individuals to comply as best they can to the recommendations or instructions provided by the researchers. Those who disengage prematurely are likely to have poorer treatment outcomes. Hence, a better understanding and an added component, such as engagement, is vital to the development of successful compliance rates. Technology, such as the mobile-based Ecological Momentary Assessment (mEMA), has been used by researchers to collect data on participants through their smartphones. Studies have also used social media and mEMA in the past for topics such as chronic conditions, physical activity, weight management, and dietary behaviors. This article reports the results of two approaches to dietary monitoring using social media and mEMA technology and the lessons learned from the two studies for improving participant compliance.

## Introduction

Successfully changing dietary behavior not only requires a basic knowledge of nutrition but also the ability to cultivate motivational factors that guide self-regulatory processes (Harrigan et al., 2016; Schwarzer, 1999). Finding ways to motivate individuals to consume more fruits and vegetables and adhere to healthy eating and food preparation are examples of self-managed health practices (Hu, Huang, Wang, Zhang, & Qu, 2014); however, poor dietary habits are difficult to change, and various social, psychosocial, and environmental factors are associated with such shifts in behavior. One has to form an explicit behavioral intention (Schwarzer, 2008), and intentions often fail to translate into corresponding behaviors. Therefore, intentions must be supplemented with other factors. For instance, an interactive engagement (i.e., reinforcement) may facilitate the translation of intentions into action and increase compliance (Cotter, Durant, Agne, & Cherrington, 2014). Reinforcement may include incentives to spur a behavioral change, which could increase the likelihood that the behavior will be repeated, become habit, and increase compliance (Kodak, Miltenberger, & Romaniuk, 2003).

Compliance can be defined as the extent to which a person’s behavior corresponds with agreed recommendations (Burgess, Hassmén, & Pumpa, 2017). Within the context of lifestyle intervention, this may refer to physical activity, diet modifications, or lifestyle intervention program attendance or attrition (Burgess et al., 2017). Regarding lifestyle intervention programs, it is crucial for individuals to comply as best they can to the instructions provided by the researchers to increase success (as measured against a variety of health and lifestyle markers). Those who disengage prematurely are likely to have poorer treatment outcomes. Hence, a better understanding and an added component, such as engagement, is vital to the development of successful compliance rates.

With notable exceptions, few studies have focused specifically on strategies to improve dietary compliance using technology (Collins, Morgan, Warren, Lubans, & Callister, 2011). The influence of social network and social engagement has become the focus of much research as social media continues to alter the way people interact with one another. Social networks can trigger mechanisms that result in increased self-efficacy (Burke, Conroy et al., 2011).

Technology, such as the mobile-based Ecological Momentary Assessment (mEMA) application, has been used by researchers to collect data on participants through their smartphones. Studies have used social media and mEMA in the past for topics such as chronic conditions, physical activity, weight management, and dietary behaviors (Bruening, Ohri-Vachaspati et al., 2016; Bruening, Van Woerden et al., 2016; Spook, Paulussen, Kok, & Van Empelen, 2013). Of the topics mentioned, dietary behavior has proven the most challenging in terms of participant compliance rate, usability, and engagement.

This article reports the results of two approaches to dietary monitoring using social media and mEMA technology, to include the lessons learned from the studies for improving participant compliance. The two studies were conducted by the same research team, and we compare the compliance rate of the two studies involving the use of technology. Choosing the proper research design to increase compliance is the basis of this article as well as the reason for comparing the two studies.

Comparison explicitly contrasts two or more cases to explore parallels and differences. Frequently, these cases are compared with regard to a specific phenomenon, such as compliance in the use of technology in dietary interventions. More often than not, the main goal is to arrive at a typology based on the observed differences and similarities among cases, even though enhancing knowledge of singular cases is a major purpose in many comparative studies. The justification is that, given the importance and spread of comparison as a research method and given the lack of sufficient attention paid to various methodological issues when conducting technology in dietary interventions that concern comparison, this article sets out to present and discuss aspects of the question using the concept of compliance.

The purpose of the first study was to examine the feasibility, acceptability, and efficacy of using Facebook as a platform to document and promote health interventions. The purpose of the second study was to investigate the feasibility of mEMA’s new photo-capturing feature to analyze the participant’s dietary intake. Due to practical constraints, this article cannot provide a comprehensive analysis of the quality of food in both studies, but this article will report on participant compliance in both studies. The major aim of this study was to report participant compliance in dietary self-management (i.e., recording food intake) by comparing the results of participants using a social media platform to those using mEMA. In doing so, our intent was to recommend new approaches to increase compliance using mobile technology and social media for future dietary intervention studies.

## Study I

The interactive nature of social media may have a different influence on a culture than non-interactive technologies (e.g., television, movies, and music), even in terms of diet. Researchers have identified that social interaction can influence food choices and portions (McFerran, Dahl, Fitzsimons, & Morales, 2010). For example, an experimental study concerning food consumption in social situations reported that participants who followed a thin consumer in a food line took significantly less food than participants who followed an obese consumer (McFerran et al., 2010). If social situations can influence behavior, social media may have a similar effect. Food consumers use social media to inform others about what they eat through posts, restaurant reviews, recipes, and pictures, and social media also affords users the opportunity to control how they present themselves to their social network.

### Methods

This feasibility study evaluated the potential of an innovative social media intervention. Twenty Filipino Americans (*N* = 20) who self-reported a chronic disease were recruited from a primary physician’s office and enrolled in a one-week study after the clinic and the Institutional Board (IRB) of the University of Nevada, Las Vegas (UNLV), approved the study. The inclusion criteria were Filipino Americans, 18 years of age or older, who self-reported a chronic disease; were currently active and/or experienced social network (Facebook) users; and could speak, read, and understand English and/or Tagalog. Active social media users were defined as individuals who had at least one existing social media account (i.e., Facebook, Twitter, Instagram) and accessed their social media account(s) at least once a week.

The exclusion criteria were (a) respondents who did not speak, read, or understand English and/or Tagalog; (2) respondents who were pregnant or diagnosed with a medical condition that required a prescribed therapeutic diet; (3) Filipino Americans with less than one year of residence in the U.S. who may not have had enough experience to identify dietary changes since immigrating to the U.S.; (4) respondents who were vegetarians; and (5) Filipino Americans who did not own a smartphone and were not experienced social network users.

As this was a pilot study with no prior information to base a sample size, we followed the recommended sample size of 12 participants per group. Our final enrollment was 20 participants, which exceeded the minimum requirement.

Participants were enrolled in a closed, private (secret) group on Facebook. A secret group was preferred over a closed group environment because of its increased security measures. For closed groups, only members of the group can see posts; however, closed groups are discoverable on Facebook, and a Facebook user who is not part of the closed group can identify and determine Facebook users who are members of the closed group. In contrast, a Facebook secret group is an area on Facebook where *only members can find the group and see group posts and membership*. This arrangement provided more privacy protection for the study participants and the facilitator. Participants also received direct messages on Facebook three times during their week of participation to encourage compliance with the study aims.

Participants were engaged in two activities during the one-week study: (1) self-monitoring of meals (i.e., photographing meals using their phone, then uploading the pictures to Facebook) and (2) participating and engaging on the study’s Facebook group page. Participants were instructed to post pictures of the food they intended to eat before they consumed it and provide a short description of the food and the preparation method (i.e., fruit and vegetable dishes, Dietary Approaches to Stop Hypertension [DASH] recipe, non-fried foods). Participants were also encouraged to post the recipe for the meal, identify where they obtained the ingredients, and engage with group posts (e.g., "like" pictures that others posted or post comments).

### Results

The demographics for the Facebook study participants are shown in Table 1.

**Table 1:** Demographics of Participants in Study I

Demographics	*n* (%)
Gender	
Male	6 (30.0)
Female	14 (70.0)
Education	
High school or less	6 (35.0)
College	11 (50.0)
Graduate level	3 (15.0)
Marital status	
Single	5 (25.0)
Married	15 (75.0)
Have serious chronic illnesses	
Yes	18 (90.0)
No	2 (10.0)

*Note:* *N* = 20. Age, range 25–55; *M* (*SD*) = 40.10 (8.16).

After one week, screen captures from the group Facebook wall (i.e., messages, images, and files uploaded to the Facebook group) were coded and analyzed using Dedoose Software (SocioCultural Research Consultants, CA, USA). Dedoose provided an opportunity to evaluate the content of participant conversations. Of the 20 participants, more than half (60%) found the social media environment *very helpful* or *extremely helpful* for self-monitoring, and 70% *preferred* using Facebook to track their meals (*Yes/No*). Approximately one third (33%) found it at least *somewhat easy* to remember to take pictures. Fifteen participants (75%) demonstrated perfect daily compliance for posting pictures at least once per day during the one-week study. Thirteen (65%) participants engaged in the group at least once per day by either liking posts or commenting on posts from other participants. Eleven (55%) participants reported that engaging with others contributed to their compliance, and 25% (*n* = 5) reported that the facilitator’s direct message reminder motivated them to take pictures of their food and post to the Facebook group. The remaining 20% (*n* = 4) reported that a self-reminder encouraged them post. We also conducted a content analysis of responses during participant exit interviews on their overall experience using Facebook. Prior to conducting the main analyses, all variables in the Facebook study were screened for inconsistent or abnormal values. Missing data rates were evaluated using maximum likelihood estimation of scattered missing responses. In addition, reasons for withdrawal from the study and loss to follow-up were also charted.

We clustered common themes from the exit interviews regarding a participant’s positive experiences during the intervention. The common themes included the following:

- the ability to appeal and cater to people who do not wish to attend face-to-face sessions;
- the ability to have immediate access to a group, regardless of the geographic separation of users;
- the ability of technology—specifically the use of the smartphone camera—to capture food photos to make documentation easier;
- the concept of anonymity, which may be preferable to those who face stigma or are reluctant to disclose sensitive information in person; and
- the ability to interact with others with a similar goal, which can motivate individuals to post pictures and complete the study.

### Discussion

The study successfully demonstrated that Facebook can be used with relative ease and at a reasonable cost for a dietary self-management intervention. In some respects, these results seem to be consistent with other studies that used Facebook for the promotion of wellness and quality of life (Valle, Tate, Mayer, Allicock, & Cai, 2013; Young & Rice, 2011) in terms of participant compliance rates. Due to the short duration of the study, we did not evaluate the physiological outcomes in terms of anthropometric measurements or body mass index (BMI) of participants; however, our success rate for completion was high.

Several factors can explain the high compliance rates observed in this study. First, Filipinos—per capita—lead Facebook use on a global scale. Social media survey reports indicate Asian Americans are the predominant online users of social networking sites worldwide, and in a survey among the top 10 nations using Facebook, Filipinos were reported as the highest-ranked Facebook users at 93.9% (Bender et al., 2014). Second, the community environment created by our participants may have encouraged them to engage with each other (e.g., liking posts or posting comments) and contributed to compliance. Consistent with the literature, Young and Rice (2011) conducted a randomized control study among men who have sex with men (MSM) and showed that participants who had a social media account (specifically Facebook) and interacted with other study participants were highly satisfied, engaged in the study, and were more likely to finish the intervention. It is also worth mentioning that one of the themes that emerged during the exit interviews was the participants’ ability to interact with others who shared similar goals, which motivated them to post pictures and complete the study. These findings provide some support for the conceptual premise that engagement can contribute to compliance. However, more research on this topic using a larger randomized study must be undertaken before the association between engagement and compliance is clearly understood.

## Study II

A healthy diet is one of the recommended strategies to prevent chronic illnesses—to include cardiovascular diseases, diabetes, and cancer (Must et al., 1999)—and healthy adults are facing with the challenge of managing and tracking their diets (Champagne et al., 2002). Traditional food journaling can be time consuming and is subject to low adherence (Bruno & Silva Resende, 2017), and this method requires recall memory, which may lead to inaccuracies and underreporting of food intake (Cordeiro et al., 2015). Therefore, the second study explored the feasibility of using the real-time photo capture and data entry capabilities of the mEMA application to analyze dietary intake based on participant photo logs. These preliminary results will only focus on the feasibility of the application features and participant compliance using mEMA technology. We enrolled 20 participants in our study and tested the effect of this feature over a one-week period.

### Methods

Upon approval from the UNLV IRB, participants were recruited from a public university located in the United States. The recruitment was completed by a trained research assistant. Out of the 20 students who were approached to participate in the mEMA study, 20 of 20 students (100%) participated for 6 consecutive days. Participants were asked to (1) download and install the mEMA application on their smartphone and (2) record their food intake (i.e., food types and amounts for breakfast, lunch, dinner, snacks, and supplements) by taking a photo of their food items using the mEMA app. Participants were then asked to synchronize the data, both text and photos related to their intake, from their smartphones to the mEMA server daily. To protect the privacy of the participants, all participants were coded. Personally identifiable information (e.g., name, phone numbers, e-mail) was kept separate from the participant codes in a password-locked computer and was not linked to the data uploaded into the mEMA app. Only approved research team members had access to this data.

Data was extracted from the server onto the researcher’s computer and analyzed using SPSS version 25. At the end of Day 6, participants were asked to answer open-ended questions regarding the use of the mEMA app for tracking food intake. This study only reports the data from breakfast, lunch, and dinner entries.

Demographics for Study II participants are listed in Table 2.

**Table 2:** Demographics of Participants in Study II

Demographics	*n* (%)
Gender	
Male	4 (20)
Female	16 (80)
Race/ethnicity	
White	8 (40)
Hispanic, Latino	1 (5)
Asian	8 (40)
Mix	3 (15)
Education	
High school or less	1 (5)
College	17 (85)
Graduate level	2 (10)
Marital status	
Single	16 (80)
Married	5 (20)
Have serious chronic illnesses	
Yes	–
No	20 (100)

*Note:* Age, range 20–46; *M* (*SD*) = 26.45 (7.29).

### Results

Participant compliance was reported for each day through the mEMA application (*N* = 20). Nineteen participants started using the mEMA app on Day 1 (95%), and 6 days later, 13 participants (65%) were still engaged and taking food pictures at least once per day. The overall compliance rate for taking food pictures or logging food items on the mEMA app at least once per day was 78%. Interestingly, the mEMA app was used 23 times without the participant being prompted. Total response rates were assessed by calculating the percentage of answered prompts per day. From Day 1 through Day 6, total response rates were 65%, 63%, 60%, 50%, 50%, and 35%, respectively (*n* = 60 prompts per day).

Participants reported that the photo feature of the mEMA app was useful, and they enjoyed the ability to monitor their food intake using both photos and text. However, most participants reported technical difficulties with the mEMA app, especially as the app functions were inconsistent across phone types (e.g., Android vs. iPhone). In addition, the app only allowed participants to take one food photo at a time, and participants felt that it was time consuming to transfer the food photos.

### Discussion

We tested the feasibility of using the mEMA application to capture participant food intake in one week. These results further demonstrate a higher use rate for mEMA app compared to the online version of the Automated Self-Administered 24-hour (ASA24) dietary recall in assessing participant food and beverage intake over a seven-day period (Bruening, Van Woerden et al., 2016). This result may be explained by the fact that our participants were younger and part of the first generation to grow up using mobile technologies.

The results of our study are significant in at least one major aspect concerning compliance. The use of mEMA could lead to decreased participant burden, yielding higher rates of compliance and lower rates of missing data in dietary self-management studies. Another possible explanation for our findings is that the positive compliance rate is due to the fact that mEMA technology capitalizes on real-time or near-real-time results in the contexts in which they occur (Ashurst et al., 2018). The same study also accords that younger individuals preferred using real-time technology features that would allow them to multitask and exercise their privacy. Overall, our participants’ compliance rate for using mEMA technology was lower than the Facebook study.

## Overall Discussion

The two studies examined the use of technology to capture participant food intake and track compliance. The first study used Facebook to display the food pictures and generate engagement among users of that particular technology. The second study used the smartphone camera as part of the mEMA app. The two studies relied on publicly available technology, which offered a low-cost model that participants could replicate and continue to use.

Overall, our findings suggest that mobile technology is an acceptable and feasible way to document food intake for the two populations studied. However, the findings also highlighted that, even under conditions in which self-monitoring was overseen by researchers with access to the food photo journals, compliance decreased over the span of the intervention in the second study. We compared compliance rates between the two studies, and our results suggest that engagement could be a factor in the higher compliance rate of the first study compared to the second study. Therefore, one of the challenges in designing mobile interventions resides in developing the capacity to integrate and sustain engagement over the period of intervention.

Feasibility was examined in multiple ways using daily metrics, to include a binary indicator of compliance for the photo-posting protocol and a count indicator for the total number of posted pictures per day. In addition, we computed overall person-level summaries of compliance for both measures. These methods present advantages and disadvantages in the context of our study, so we chose to consider them concurrently. Intervention compliance was broadly conceptualized as posting food pictures, as requested by the researchers. However, since participants were not required to eat a certain number of times a day but rather document their typical eating habits in both studies, we could not establish an expected number of posts per day. Therefore, we first chose to capture a global dichotomous metric of daily compliance as it was anticipated that all participants would eat at least once a day. We also imagined that most participants would eat more than once a day, and the daily count of photo posts reflected the second component of compliance.

Both indicators revealed that compliance was good for the study overall. Over 420 photos were collected during the first study. Over 500 photos were collected during the second study—approximately two per day from each participant—indicating that this was a feasible and acceptable way to track eating behaviors. This number did fall a little below what one may expect in the hypothetical case of three meals a day, even given the disorganization that can characterize student eating patterns in the second study. Nevertheless, using the dichotomous indicator of compliance, we found that compliance declined over the course of the study with every additional study day increasing the chance of noncompliance by 10%. Furthermore, none of our baseline indicators were associated with variations in this decline. Using the total number of pictures posted as a count measure for compliance, we also found an overall decrease throughout the course of the study. Similar trends have been found in studies requiring participants to document their food and drink intake during the day.

Decrease in compliance over the course of an intervention may result from participants’ self-regulation, leading to the perception that they no longer need to adhere to their record (Barlow, Wright, Sheasby, Turner, & Hainsworth, 2002). While this is a potential explanation for our findings, it is also possible that participants progressively disengage once the novelty of a study wears off. Ways of preventing this decline may include providing personalized feedback and reinforcement for engaging in self-monitoring, potentially through peer-to-peer feedback. Another approach may be to develop gaming aspects within interventions, allowing participants to accrue points or rewards for sustained compliance. Future studies should explore the use of incentives as a means to encourage compliance.

In previous studies, using photos to self-monitor dietary intake was preferred over a written journal as aspects such as portion size or color diversity can be more easily perceived in an image (Burke et al., 2012; Burke, Wang, & Sevick, 2011). However, such methods require participants to remember to document their eating in real time rather than retrospectively, which may place a greater burden on individuals. Nevertheless, real-time monitoring is likely to be more accurate than recall.

Based on our findings, we have tentatively formulated a few recommendations regarding the design of interventions that seek to capitalize on mobile technology to promote healthy eating. First, it seems that online messages—even at a frequency of three times a day—were well-received by participants. Furthermore, participants expressed that they would welcome specific messages that remind them to complete their self-monitoring tasks. Second, while Photo App was a free phone application used in the study, participants preferred to use a more familiar mainstream app with similar functions that is more culturally congruent. Third, when possible, it may be helpful to either define study expectations more narrowly (e.g., number of entries in the photo food journal) or obtain an additional measure of behavior to compare the number of entries to the number of behavior occurrences (e.g., meals eaten but not posted). Finally, it could be useful to develop a food photo journal app in a future study to increase the ecological validity of results and guide the development of evidence-based tools as participants may be more likely to choose such an application for their personal use.

Many of our findings point to the need to regard compliance as a multidimensional concept that involves different perspectives, methods of observations, and levels of assessment that can be context-bound, sociocultural variables. Individuals may not comply with recording food intake, even with the use of technology such as mEMA, or they may comply with food recording but refuse to do so to a different degree in a different situation and during a longer intervention. Personal reasons for noncompliance in recording food intake via technology may be understandable (e.g., did not remember to take pictures due to time constraints) once those reason are identified. However, the addition of social engagement and interaction using technology could increase compliance as in the case of the Facebook study.

### Limitations

Our study has several limitations. First, the interventions were relatively short, and one week may be insufficient for developing new habits and behaviors. Therefore, it would be important to examine whether compliance would have continued to decline beyond the one-week period. Second, in the first study, all participants had some experience with Facebook, and peer engagement was not a factor we injected into the study. More personalized intervention content and/or feedback may help improve intervention adherence in the second study, such as the use of rewards, points, or games. Finally, we only recruited participants possessing a phone with Internet access, which may have biased the sample. Other authors have commented on the "digital divide" and how an increasing gap exists between individuals who possess access to and the skills to use the Internet and social media and those who do not. Given this divide is associated with socioeconomic factors, it may increase disparities in terms of health. Future studies should keep this divide in mind and consider how to minimize these factors.

### Nursing Implications

Nurse scientists who use technology in their research must collaborate with their target communities, so choosing to integrate mobile technologies into community-based research can be beneficial for the community as well as for future research, service provision, and practice. The effectiveness of any technology-based research intervention is directly related to the ability of that platform to increase or sustain participant compliance through engagement. Clearly, the use of Facebook is related to engagement in the real world, and it is possible that researchers could develop platforms with features similar to Facebook that maximize both participant engagement and compliance rate. Indeed, the results of our Facebook study support this notion: that technologies participants already use can be repurposed in ways that improve dietary self-monitoring within the context of high rates of compliance and, eventually, positive outcomes.

### Conclusion

Our findings suggest that interventions capitalizing on mobile technology through the use of photo journals for self-monitoring are acceptable and feasible among emerging adults, which supports the development and further evaluation of commercially available tools. Additional research is warranted to determine how to increase compliance through user engagement over longer time periods and to determine how the presence of personalized or interactive features may enhance intervention designs.

## Acknowledgments

The authors acknowledge the following in assisting the PI for data analysis: Denise Warner and Tanya Cooper.

## Declaration of Conflicting Interests

The author(s) declared no potential conflicts of interest with respect to the research, authorship, and/or publication of this article.

## Funding

This research was funded by University of Nevada, Las Vegas School of Nursing Intramural Grant and the University of Nevada, Las Vegas School of Nursing Center for Biobehavioral Interdisciplinary Science.
